# Cannabidiol Application Increases Cutaneous Aquaporin-3 and Exerts a Skin Moisturizing Effect

**DOI:** 10.3390/ph14090879

**Published:** 2021-08-30

**Authors:** Nobutomo Ikarashi, Marina Shiseki, Ryotaro Yoshida, Keito Tabata, Rina Kimura, Tomofumi Watanabe, Risako Kon, Hiroyasu Sakai, Junzo Kamei

**Affiliations:** 1 Department of Biomolecular Pharmacology, Hoshi University, 2-4-41 Ebara, Shinagawa-ku, Tokyo 142-8501, Japan; s171108@hoshi.ac.jp (M.S.); m2119@hoshi.ac.jp (R.Y.); s171148@hoshi.ac.jp (K.T.); s171077@hoshi.ac.jp (R.K.); rvimdkamagk@gmail.com (T.W.); r-kon@hoshi.ac.jp (R.K.); sakai@hoshi.ac.jp (H.S.); 2 Juntendo Advanced Research Institute for Health Science, Juntendo University, 2-1-1 Hongo, Bunkyo-ku, Tokyo 113-8421, Japan

**Keywords:** cannabidiol, aquaporin-3, skin, *Cannabis sativa*, cosmetics, moisturizing effect

## Abstract

Cannabidiol (CBD) is a major nonpsychotropic component of *Cannabis sativa* with various pharmacological activities. In this study, we investigated the skin moisturizing effect of CBD and its mechanism. A 1% CBD solution was applied daily to skin of HR-1 hairless (Seven-week-old, male) for 14 days. The dermal water content in CBD-treated mice was significantly increased compared to that in the control group. Furthermore, no inflammatory reaction in the skin and no obvious skin disorders were observed. The mRNA expression levels of loricrin, filaggrin, collagen, hyaluronic acid degrading enzyme, hyaluronic acid synthase, ceramide degrading enzyme, and ceramide synthase in the skin were not affected by the application of CBD. However, only aquaporin-3 (AQP3), a member of the aquaporin family, showed significantly higher levels in the CBD-treated group than in the control group at both the mRNA and protein levels. It was revealed that CBD has a moisturizing effect on the skin. In addition, it is possible that increased expression of AQP3, which plays an important role in skin water retention, is a contributor to the mechanism. CBD is expected to be developed in the future as a cosmetic material with a unique mechanism.

## 1. Introduction

The skin is an organ that exists at the boundary between the external environment and the internal environment of a living body. Dermal water content is related to the elasticity of the skin and the formation of wrinkles. In fact, in elderly individuals, dermal water content is reduced, elasticity is reduced, and wrinkles form [1]. Dermal water content is controlled by various moisturizing factors, such as collagen [2], ceramide [3,4], and hyaluronic acid [5,6]. Moreover, loricrin [7] and filaggrin [8] are involved in the barrier function of the skin and are also important for skin moisturization. In addition to these factors, it has become clear that the water channels known as aquaporins (AQPs) play important roles in skin water retention. In particular, AQP3 is predominantly expressed in the cell membrane of skin keratinocytes, and it has been reported that AQP3-knockout mice have dry skin and reduced skin elasticity compared to wild-type mice [9,10]. Moreover, cutaneous AQP3 are implicated in the development of several skin diseases, such as eczema, diabetic xeroderma, psoriasis, hyperproliferative skin disorders, or melanoma, among others, and are very important molecule that controls skin function [11]. Currently, various substances and natural products that increase these moisturizing factors and AQPs have been found and are expected to be used as cosmetic materials.

Cannabidiol (CBD) is a major nonpsychotropic component of *Cannabis sativa*. CBD is known to exert various pharmacological actions, such as anxiolytic [12], antipsychotic [13], anti-inflammatory [14], antiemetic [15], and antidiabetic actions [16], and active research is being conducted. CBD has also been found to have effects on atopic dermatitis, pruritus, and acne [17,18], and research on the effects of CBD on the skin is being conducted. Moreover, it has been reported that when CBD is applied to skin, dermal water content and the elasticity of the skin are increased [19], and there is a protective effect against ultraviolet rays [20]. As mentioned previously, CBD is attracting attention as a cosmetic material. However, scientific evidence is still scarce, and it remains unclear how CBD affects the genes that regulate skin function.

Therefore, in this study, we aimed to generate scientific evidence on the usefulness of CBD by investigating the skin moisturizing effect of CBD. Specifically, CBD was applied to the skin of hairless mice, and dermal water content was measured. We also investigated the effect of CBD on the expression of genes (loricrin, filaggrin, collagen, hyaluronic acid degrading enzyme, hyaluronic acid synthase, ceramide degrading enzyme, ceramide synthase, and AQPs) involved in the maintenance of dermal water content.

## 2. Results

### 2.1. Skin Tissue Findings and Inflammatory Reactions

**Figure 1:**
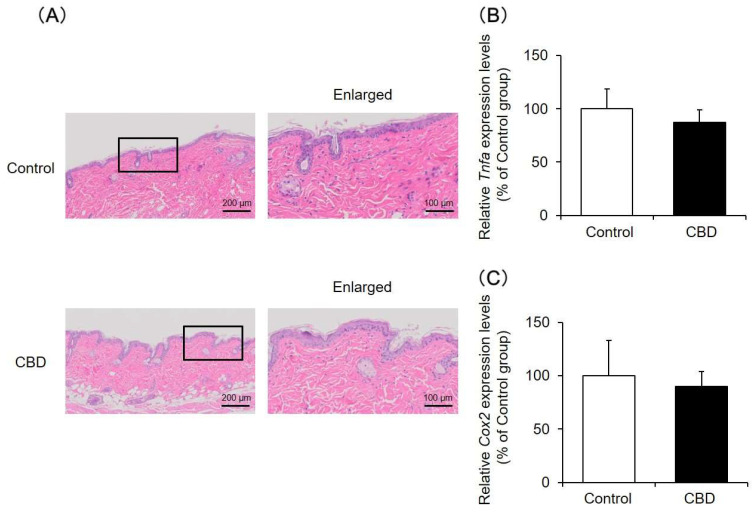
Skin tissue findings and inflammatory reactions. A 1% CBD solution was applied to the back skin of mice for 14 days. After the treatment, the skin was removed, and the tissue was assessed by HE staining (**A**). The mRNA expression levels of *Tnfa* (**B**) and *Cox2* (**C**) in the skin were analyzed by real-time RT-PCR (*n* = 6, means ± SD).

Skin tissue was analyzed after the application of CBD for 14 days by hematoxylin and eosin (HE) staining. The results showed that, even when CBD was applied, the skin tissue was similar to that in the control group, and no obvious skin tissue disorders were observed (Figure 1A). In addition, the mRNA expression levels of *Tnfa* and *Cox2*, which are increased during inflammation, were not altered by the application of CBD (Figure 1B,C).

Based on the above results, no significant skin toxicity was observed at the CBD treatment concentration in this experiment.

### 2.2. Dermal Water Content and Transepidermal Water Loss (TEWL)

**Figure 2:**
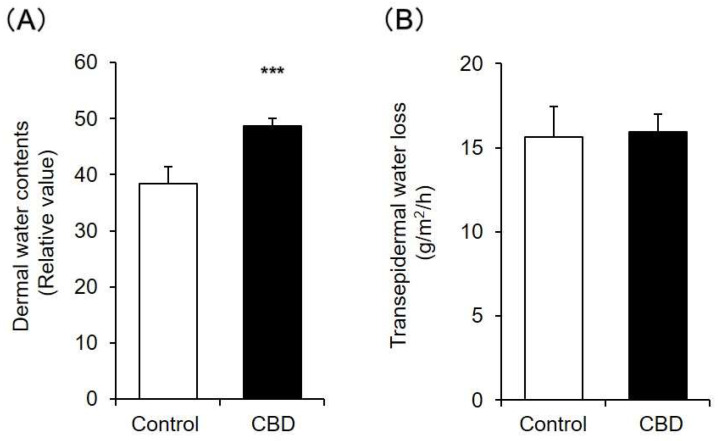
Dermal water content and TEWL. A 1% CBD solution was applied to the back skin of mice for 14 days. After the treatment, dermal water content (**A**) and TEWL (**B**) were measured (*n* = 6, means ± SD, *** *p* < 0.001 vs. the control group).

Dermal water content and TEWL after CBD was applied for 14 days were measured. The dermal water content in the CBD-treated group was significantly increased compared to that in the control group (Figure 2A). In contrast, TEWL in the CBD-treated group was similar to that in the control group (Figure 2B).

In general, when the barrier function of the skin is decreased, TEWL increases. Therefore, these results showed that when CBD was applied to mouse skin for 14 days, dermal water content increased while normal barrier function was maintained.

### 2.3. Expression Levels of Skin Moisturizing Factor-Related Genes

Dermal water content is regulated by loricrin, filaggrin, collagen, hyaluronic acid, ceramide and other factors. Therefore, whether the expression levels of genes involved in this process were changed by CBD application was investigated.

**Figure 3:**
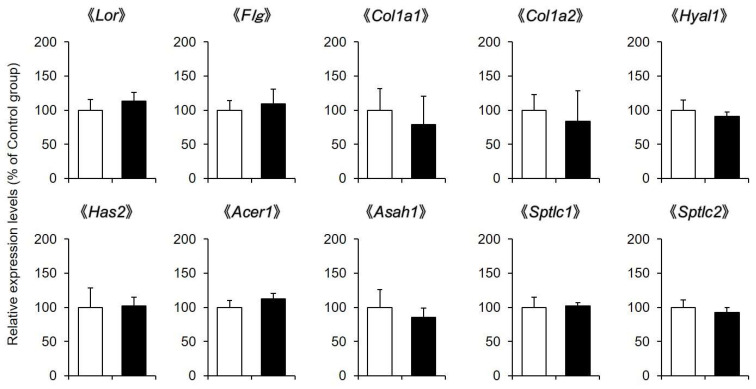
Expression levels of skin moisturizing factor-related genes. A 1% CBD solution was applied to the back skin of mice for 14 days (black). A solvent was applied to the control group (white). After the treatment, the mRNA expression levels of *Lor*, *Flg*, *Col1a1*, *Col1a2*, *Hyal1*, *Has2*, *Acer1*, *Asah1*, *Sptlc1*, and *Sptlc2* in the skin were analyzed by real-time RT-PCR (*n* = 6, means ± SD).

The mRNA expression levels of *Lor* and *Flg* in the skin after the application of a 1% CBD solution for 14 days were almost the same as those in the control group. The expression level of collagen (*Col1a1* and *Col1a2*) was not different between the CBD-treated group and the control group. Furthermore, hyaluronan degrading enzyme (*Hyal1*), hyaluronan synthase (*Has2*), ceramide degrading enzymes (*Acer1* and *Asah1*), and ceramide synthases (*Sptlc1* and *Sptlc2*) were not affected by the application of CBD (Figure 3).

### 2.4. AQP mRNA and Protein Expression Levels in the Skin

In mammals, 15 isoforms of AQPs are expressed [21] and involved in intracellular and extracellular water transport (in mice, AQP10 is a pseudogene [22]). Therefore, whether the expression level of AQP was altered in the skin after the application of a 1% CBD solution for 14 days was investigated.

**Figure 4:**
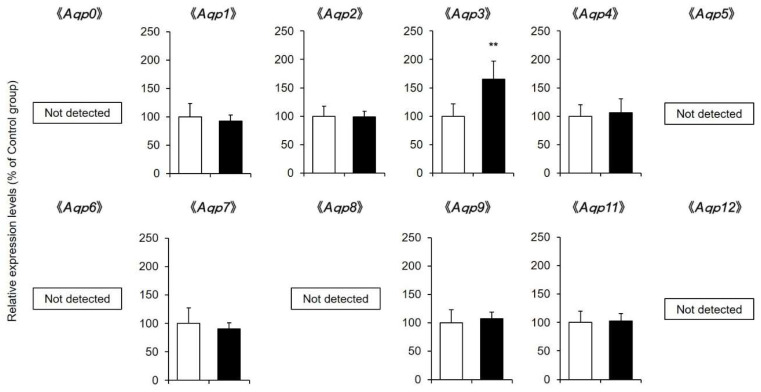
*Aqp* mRNA expression levels in the skin. A 1% CBD solution was applied to the back skin of mice for 14 days (black). A solvent was applied to the control group (white). After the treatment, the mRNA expression levels of *Aqp* in the skin were analyzed by real-time RT-PCR (*n* = 6, means ± SD, ** *p* < 0.01 vs. the control group).

Comprehensive analysis of *Aqp* mRNA expression levels by real-time RT-PCR showed that *Aqp1*, *Aqp2*, *Aqp3*, *Aqp4*, *Aqp7*, *Aqp9*, and *Aqp11* were present in mouse skin. Of these, only the mRNA expression level of *Aqp3* was confirmed to be significantly increased in the CBD-treated group compared with the control group (Figure 4).

**Figure 5:**
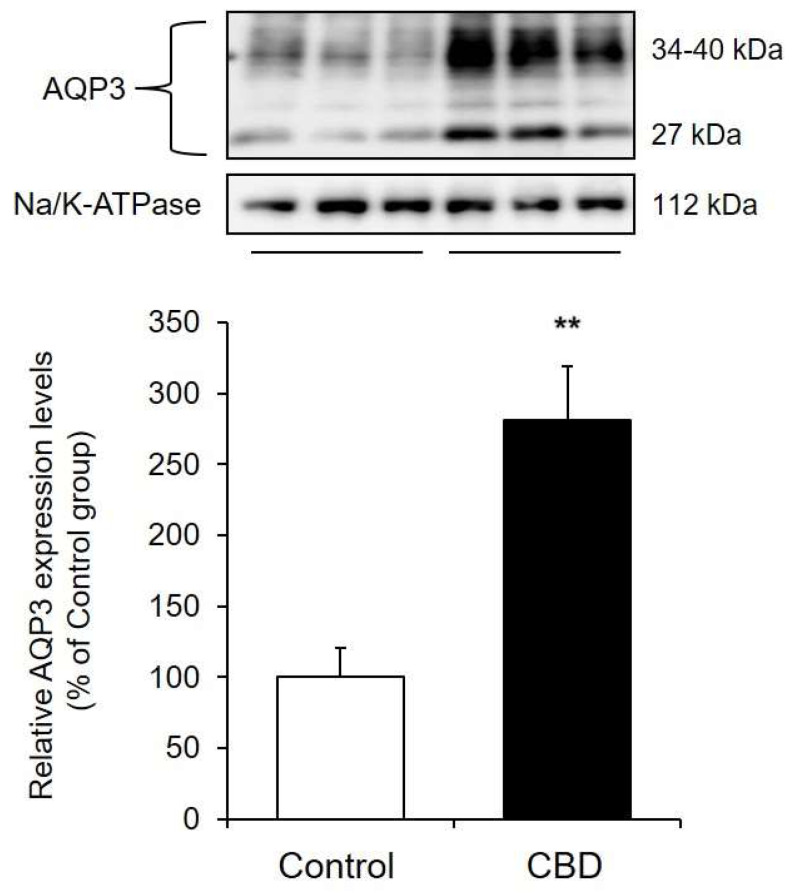
AQP3 protein expression levels in the skin. A 1% CBD solution was applied to the back skin of mice for 14 days. After the treatment, the protein expression level of AQP3 in the skin was analyzed by Western blotting (*n* = 6, means±SD, ** *p* < 0.01 vs. the control group).

When the protein expression level of AQP3 was analyzed by Western blotting, two bands were detected. The band at approximately 27 kDa was nonglycosylated, and the band at approximately 30–40 kDa was glycosylated. Since there was no difference in water permeability between nonglycosylated AQP and glycosylated AQP [23,24,25], the total of these band densities was used for analysis in this study. The protein expression level of AQP3 after CBD treatment was significantly higher than that in the control group (Figure 5).

These results showed that CBD enhanced the transcription of *Aqp3* in the skin and increased the protein expression level of AQP3.

## 3. Discussion

CBD has been reported to have improving effects on various skin diseases [17,18,19,20]. In this study, we verified the skin moisturizing effect of CBD and analyzed its mechanism.

Laura Casares et al. have shown that the application of a 1% CBD solution to the skin of 6-month-old mice induces keratinocyte proliferation, which may be involved in wound healing [26]. Therefore, as in a previous report, a 1% CBD solution was applied to mouse skin every day, and various examinations were conducted. The results showed that dermal water content in the CBD-treated group increased approximately 1.3 times compared with that in the control group, and a significant difference was observed (Figure 2A). Moreover, no skin inflammation was observed (Figure 1), and the skin barrier function was normal (Figure 2B). These findings show that CBD exhibits a skin moisturizing effect and may be considered useful as a cosmetic material. When a 3% CBD solution was applied to mouse skin, no inflammatory reaction was observed, but TEWL increased and dermal water content decreased to the same level as in the control group (data not shown). Therefore, an optimal concentration was considered when applying CBD to the skin.

Dermal water content is regulated by moisturizing factors such as loricrin, filaggrin, collagen, hyaluronic acid, and ceramide. Therefore, we investigated the effect of CBD on the expression of genes involved in these processes. CBD did not affect the mRNA expression levels of loricrin, filaggrin, collagen, hyaluronan degrading enzyme, hyaluronan synthase, ceramide degrading enzyme, or ceramide synthase in the skin (Figure 3). Therefore, it is unlikely that CBD exerted a skin moisturizing effect by changing the amount of these moisturizing factors.

In recent years, it has been reported that the water channel known as AQP plays an important role in skin water retention [9,10], and active research is being conducted. AQP is a water-permeable protein expressed on the cell membrane, and 15 isoforms of AQPs are known to exist in mammals. *Aqp1*, *Aqp2*, *Aqp3*, *Aqp4*, *Aqp7*, *Aqp9*, and *Aqp11* mRNA were detected in the skin of the mice in this study (Figure 4). Of these, it was revealed that only the mRNA expression level of *Aqp3* was significantly increased by the application of CBD compared with that in the control group (Figure 4). The protein expression level of AQP3 was also significantly higher in the CBD-treated group than in the control group (Figure 5). AQP3 is predominantly expressed in the cell membrane of skin keratinocytes and is classified as an aquaglyceroporin, which is responsible for transporting glycerol as well as water [27]. It has been reported that AQP3-knockout mice have dry skin and reduced skin elasticity compared to wild-type mice [10]. In addition, the expression level of AQP3 in the skin of aged mice was lower than that in young mice, suggesting that AQP3 may be involved in the decrease in dermal water content in old age [28]. Furthermore, AQP3 is decreased at the onset of various pathological conditions (diabetes mellitus [29,30], vitiligo [31,32], and psoriasis [33]) in which dry skin is observed. In addition, when mice are treated with the anticancer drug, which causes marked dry skin as a side effect, dry skin and a decrease in AQP3 were also observed [34]. Thus, it can be hypothesized that AQP3 is a key molecule that plays an important role in skin water retention. Since CBD increased the expression of AQP3 in the skin, it was thought that this effect may be involved in the skin moisturizing effect of CBD.

AQP3-induced glycerol transport contributes not only skin water retention but also to skin protection. AQP3 facilitates glycerol movement in several cells of skin layers, including the epidermal keratinocytes [10,35] or hypodermal adipocytes [36]. It was reported that topic or systemic glycerol administration or AQP3 re-expression in keratinocytes reverse the phenotype of skin abnormalities in *Aqp3*-deficient mice [35,37]. In this study, although the skin glycerol concentration in CBD-treated mice was not measured, it is possible that the increase in glycerol associated with the increase in AQP3 is also involved in the increase in dermal water content.

AQP3 is known to be present not only in keratinocytes but also in hypodermal adipocytes. However, its expression level is very low, and it is a fact that the epidermal layer is strongly stained when the skin is performed immunohistochemistry of AQP3 [11,28]. As AQP3 in the epidermal layer is mainly involved in the increase in dermal water content, we believe that the increase in dermal water content due to CBD treatment is mainly due to the increase in AQP3 in the epidermal layer.

Why did the application of CBD increase AQP3 in the skin? So far, various cytokines, such as tumor necrosis factor-α (TNF-α) or transforming growth factor-β (TGF-β), can change the expression levels of aquaglyceroporins AQP3, AQP7, and AQP11 in subcutaneous adipocytes [38,39]. In this study, it was clarified that the application of CBD to mouse skin did not change the cutaneous *Tnfa* mRNA expression level, and at this time, only *Aqp3* increased; no correlation was found between *Tnfa* and *Aqps* transcript levels. Therefore, it is unlikely that these cytokines are involved in the increased *Aqp3* expression.

In general, CBD acts on cannabinoid receptors to exert various pharmacological actions [40]. On the other hand, it has also been reported that CBD increases the activity of peroxisome proliferator-activated receptor-γ (PPARγ) and exerts a pharmacological effect on various cells [41,42]. PPARγ is expressed in skin keratinocytes, and it has been revealed that PPARγ agonists increase the expression level of AQP3 in keratinocytes [43]. In this study, there was no change in the mRNA expression level of *Pparg* in mouse skin treated with CBD (data not shown). Although the mechanism by which CBD increases AQP3 in mouse skin is unclear, it is possible that CBD activates PPARγ in keratinocytes and increases AQP3, which requires further study.

The results of this study show that CBD has a skin moisturizing effect. In addition, it is possible that the increased expression of AQP3, which plays an important role in skin water retention, is involved as one of the mechanisms of action. Currently, there is an active search for substances and natural products that increase cutaneous AQP3 expression. CBD is expected to be applied in the future as a cosmetic material that has the unique effect of increasing AQP3.

## 4. Materials and Methods

### 4.1. Materials

CBD crystalline powder (Batch No. CB99020171A) was provided by PharmaHemp Laboratories (Ljubljana, Slovenia) and used in this study. This powder contained 99.34% CBD.

### 4.2. Animals and Treatment

Seven-week-old male HR-1 hairless mice were purchased from Hoshino Laboratory Animals, Corp. (Ibaraki, Japan). The mice were divided into two groups (*n* = 6): a control group and a CBD-treated group. In the control group, an aqueous solution containing 10% propylene glycol and dimethyl sulfoxide was applied to the skin on the backs of the mice for 14 days. In the CBD-treated group, an aqueous solution containing 1% CBD was applied to the skin of the mice for 14 days. After the application was completed, dermal water content (Corneometer CM825; Courage & Khazaka, Cologne, Germany) and TEWL (Tewameter TM300; Courage & Khazaka, Cologne, Germany) were measured. The back skin was removed under isoflurane anesthesia, and skin tissue damage was observed by HE staining. To analyze mRNA and protein expression, the back skin was instantly frozen in liquid nitrogen and stored at −80 °C.

### 4.3. HE Staining

The skin was soaked in 4% formaldehyde solution to fix the tissue. Tissue samples were embedded in paraffin and mounted on glass slides. The slides were deparaffinized, stained with HE, and observed under a microscope. The slides were blindly evaluated by a pathologist and evaluated for skin damage.

### 4.4. Real-Time RT-PCR

Total RNA was extracted by homogenizing approximately 20 mg of skin with Tri reagent (Sigma-Aldrich Corp., St. Louis, MO, USA). cDNA was synthesized from total RNA using a high-capacity cDNA synthesis kit (Applied Biosystems, Foster City, CA, USA). The cDNA solution, primers (Table 1), and Sso advanced universal SYBR Green SuperMix (Bio-Rad Laboratories, Hercules, CA, USA) were combined, and the mRNA expression level was analyzed using a real-time PCR system. The mRNA expression level was normalized using 18S rRNA. Genes with more than 35 cycles were not detected.

### 4.5. Western Blotting

Approximately 150 mg of skin was homogenized in dissecting buffer. According to past reports [44], centrifugation was performed to prepare a sample solution that was used for Western blotting. The sample solution was diluted with loading buffer after the protein concentration was measured by the BCA method. The proteins were separated by electrophoresis and then transferred to a polyvinylidene difluoride membrane. The membrane was blocked with blocking buffer and then incubated with primary antibodies [rabbit anti-rat AQP3 (Alomone Labs, Jerusalem, Israel), mouse anti-rabbit Na/K-ATPase (Merck Millipore, Darmstadt, Germany)] and secondary antibodies [donkey anti-rabbit IgG-HRP (Santa Cruz Biotechnology Inc., Santa Cruz, CA, USA) and goat anti-mouse IgG-HRP antibody (Merck Millipore, Darmstadt, Germany). The membrane was reacted with enhanced chemiluminescent prime Western blotting detection reagents (GE Healthcare, Fairfield, CT, USA), and bands were detected by an immunoimage analyzer (ImageQuant LAS500, GE Healthcare). The protein expression level was normalized using Na/K-ATPase.

### 4.6. Statistical Analysis

The values are shown as the mean ± standard deviation (SD). Normality was tested through Shapiro-Wilk test, and homogeneity of variances tested through F test. Based on the normality of distribution and variance homogeneity, Student’s *t*-test was used for analysis, and *p* < 0.05 was considered significant.

## 5. Conclusions

It was revealed that CBD has a moisturizing effect on the skin. In addition, it is possible that increased expression of AQP3, which plays an important role in skin water retention, is a contributor to the mechanism. CBD is expected to be developed in the future as a cosmetic material with a unique mechanism.

## Figures and Tables

**Table 1:** Primer sequences.

Gene	Accession Number	Forward (5′ to 3′)	Reverse (5′ to 3′)
*Tnfa*	NM_013693	AAGCCTGTAGCCCACGTCGTA	GGCACCACTAGTTGGTTGTCTTTG
*Cox2*	NM_011198	CAGGGCCCTTCCTCCCGTAG	GCCTTGGGGGTCAGGGATGA
*Lor*	NM_008508	GCCGATGGGCTTAACTTTCT	CAGGATACACCTTGAGCGAC
*Flg*	NM_001013804	AAGGAAATCAGTCTTGCCGT	CTGACCTTCTGAGACACACC
*Col1a1*	NM_007742	CCCGAGGTATGCTTGATCTG	GGTGATACGTATTCTTCCGGG
*Col1a2*	NM_007743	TCTCACTCCTGAAGGCTCTA	GTAGTAATCGCTGTTCCACTC
*Hyal1*	NM_008317	TTTCTTTGAGCCTGGAGCTA	GTAGTTTCCTTTCGTTGGCT
*Has2*	NM_008216	CGTGGATTATGTACAGGTGTGT	CCAACACCTCCAACCATAGG
*Acer1*	NM_175731	CCGAGTTCTACAATACGTTCA	CATACGGATGCATGAGGAAC
*Asah1*	NM_019734	CTGTCCTCAACAAGCTGACTG	TCTCAGTACGTCCTCAAGGC
*Sptlc1*	NM_009269	TCCCCTTCCAGAACTGGTTAAA	CCATAGTGCTCGGTGACT
*Sptlc2*	NM_011479	GTCAGGAAATTGGAAACCTGG	AGCTTCCACACCTAAGAACC
*Aqp0*	NM_008600	TGCTCTGCATCTTTGCTACA	GCACCAGTGTAATACATCCCA
*Aqp1*	NM_007472	CTGCTGGCGATTGACTACACT	TCATAGATGAGCACTGCCAGG
*Aqp2*	NM_009699	CTGGCTGTCAATGCTCTCCAC	TTGTCACTGCGGCGCTCATC
*Aqp3*	NM_016689	CCTTGTGATGTTTGGCTGTGG	GGAAGCACATTGCGAAGGTC
*Aqp4*	NM_009700	GAGTCACCACGGTTCATGGA	CGTTTGGAATCACAGCTGGC
*Aqp5*	NM_009701	GCTGGAGAGGCAGCATTG	CACCCAAGTGTCCCATCATG
*Aqp6*	NM_175087	AGTCCATTGGATCTTCTGGGT	GTCTTGGTGTCAGGGAACAA
*Aqp7*	NM_007473	GCTTGGTCTGCTGCTTCAG	GGAACTCTGCCAGAAACTCTC
*Aqp8*	NM_007474	GGCTTCTCTGTCATTGTGGA	TCCGATGAGGAGCCTAATGA
*Aqp9*	NM_022026	TGAGCCATTAGGAGAGACCTT	ACCTCCAACTTTAGTCCACCA
*Aqp11*	NM_175105	AGTCTGACCAAGTACCATTACGA	TGCATAGGCCAAAAAGGTGAT
*Aqp12*	NM_177587	GCCTGGAGATGCGAGTGTT	GCCGACAGTTTCAGCAGAG
*18s rRNA*	NR_003278	GTCTGTGATGCCCTTAGATG	AGCTTATGACCCGCACTTAC

*Tnfa*: tumor necrosis factor-α, *Cox2*: cyclooxygenase-2, *Lor*: loricrin, *Flg*: filaggrin, *Col1a1*: collagen type I alpha 1 chain, *Col1a2*: collagen type I alpha 2 chain, *Hyal1*: hyaluronidase 1, *Has2*: hyaluronic acid synthase 2, *Acer1*: alkaline ceramidase 1, *Asah1*: N-acylsphingosine amidohydrolase 1, *Sptlc1*: serine palmitoyltransferase 1, *Sptlc2*: serine palmitoyltransferase 2.

## Data Availability

Data is contained within the article.
